# Game Theory Based Trust Model for Cloud Environment

**DOI:** 10.1155/2015/709827

**Published:** 2015-08-25

**Authors:** K. Gokulnath, Rhymend Uthariaraj

**Affiliations:** Ramanujan Computing Centre, Anna University, Sardar Patel Road, Chennai, Tamil Nadu 602001, India

## Abstract

The aim of this work is to propose a method to establish trust at bootload level in cloud computing environment. This work proposes a game theoretic based approach for achieving trust at bootload level of both resources and users perception. Nash equilibrium (NE) enhances the trust evaluation of the first-time users and providers. It also restricts the service providers and the users to violate service level agreement (SLA). Significantly, the problem of cold start and whitewashing issues are addressed by the proposed method. In addition appropriate mapping of cloud user's application to cloud service provider for segregating trust level is achieved as a part of mapping. Thus, time complexity and space complexity are handled efficiently. Experiments were carried out to compare and contrast the performance of the conventional methods and the proposed method. Several metrics like execution time, accuracy, error identification, and undecidability of the resources were considered.

## 1. Introduction

Cloud computing caters versatile service needs to diverse computing requirements. Services are catered through established TCP/IP network. Service providers and service users are distinct of each other; this raises the notion of trust. Trust is always unease for the distributed computing environment. Trust is the expected continuous behaviour of an entity. Users and resources vary over time in cloud environment; thus dynamic nature of the cloud environment makes trust even complex. Thus, trust is a major concern in cloud computing. Cloud computing faces the challenge of trust in its own way. Resources are abstracted from applications because of usage of third-party owned resources (datacenter) and question of belief arises. Cloud faces the challenge of trust in following ways. First, virtual machine (VM) IDs created during VM boot up by host machines are temporary. Hence, if anything goes wrong during execution, host machines can trace the actual VM using VMID. But, it is difficult to trace the actual VM which carried out the task after the shutdown. Thus reassigning becomes difficult. Second, Probability of a high trust rated resource failure is possible. Thus, ensuring trustworthiness for each interaction results in safer transactions and finally, due to growing popularity, new vendors getting into market to provide services. If they are utilized properly, service utilization can be increased and waiting time shall be reduced to greater extent.

In distributed computing paradigm's trust evaluation, initial trust or trust bootstrapping begins with uncertainty over resource trustworthiness. Bootstrapping of trust in cloud environment is quite uneasy; at the same moment it is vital for both users and service providers. Generally, trust in distributed system is classified into two categories, namely, indirect trust and direct trust. Indirect trust is further classified into reputation based trust and recommendation based trust. The purpose of the reputation based trust [1] is to measure trustworthiness of the resources by the credibility earned globally. Services are well explored by the global users; hence it is not mandatory for individual users to experience and test the trustworthiness. Services are obtained from accepted service providers. Levels of trust are considered high for the resources. However, new resources for rendering services may not be evaluated by this method. Reputation earned for services may vary based on the context; thus, context specificity is also to be considered. Estimated trust values are obtained from external entities and owing to this reason reliability is a concern. Furthermore, this type of evaluation may increase time complexity of trust calculation because of the complex analysis involved in analyzing the feedback [2] from global users. Recommendation based trust [3] aggregates the trust values obtained from trusted friends. Users with transaction history with a resource of interest pass the trust value calculated to other users for exploring the service. This method helps to evaluate trust without global reputation or direct past interaction. Computational complexities of identifying the friends, communication cost involved in transit, and estimating the trust acquired are the overheads to be considered. Moreover, like the reputation based category, reliability is a concern in this category, because of the contribution of external entities. Direct trust [4] measures the trustworthiness of the resource provider through the past history of transactions from self-encounters and its subsequent trust values assessed. Since the external entities involvement is reduced to a greater extent, computational complexity involved is comparatively minimal compared to the previous methods and also the communication cost is avoided. Further the history of transaction is mandatory for evaluation. The improvement of the reliability does not rely upon external entities. New requests face trouble of no past statistics; thus assessment of trust faces challenge. New requests have no past statistics and hence assessment of trust is a challenge. Since statistics-maintenance is essential for this method, it results in space complexity.

Most of the trust evaluation methods presented in literature are based on past transactional history. However, bootload level of trust evaluation is rarely identified. This work emphasizes direct trust for establishing trust relationship with cloud users and/or providers. Reliability for direct trust is comparatively higher than other discussed trust evaluations. Moreover, it should be considered how initial trust obtains the trust value for interaction. In case of uncertainty it assigns equally good level and bad level of trust. Computational complexity is considerably higher in assigning the values for uncertain sources. Response time can be considerably improved, if the computation is reduced.

In addition, as per Gartner's prediction [5], by 2016, the enterprises will make independent testing of security as mandatory proof to use any service provided by cloud. Hence, direct trust may have a major contribution towards assessing cloud services in future. This is to be handled precisely. Eventually, the proposed method addresses the issue. Furthermore, Cloud Security Alliance (CSA) report [6] says the number of cloud threat issues has grown to 12. Trust and uncertain risk profile are the serious challenges to be combated. Thus from the analysis of the current trends it can be inferred that trust is an issue of serious concern. The above problem resembles one-time game. Hence, the proposed work uses game theoretic approaches for attaining trust in initial level. One-time games are also suitable for uncertain environment and are applicable for first-time interaction. As a contribution to establish trust between users to resource provider and vice versa at bootload level, this work uses 2-player Nash equilibrium. Computational complexity is handled effectively by 2-player Nash equilibrium, which falls under PPAD-complete [7]. To the best of our knowledge this bootload level of trust evaluation in cloud environment is the first of its kind.

The rest of the paper is organized as follows.  Section 2 presents the related works to this work in the literature.  Section 3 describes the game theoretic methods used in this paper; Section 4 elaborates how trust concepts assorted with game theory. In Section 5 the results achieved in this work are discussed. Finally Section 6 highlights the achievements of this work and quotes the future enhancements to be carried out. The list of references to build the work is furnished.

## 2. Related Works

Trust is a dynamic notion, which transforms with respect to time and context. Particularly, in cloud computing, frequent changes in users and service providers are highly probable and thus results in cold start problem towards the evaluation of trust. Problem of evaluating trust at initial transaction has been addressed by several works in the literature. Like the peer-to-peer network, trusting the unexplored resource may end up with the problem of whitewashing. Most common methods of bootstrapping like [8] are to allot default values (low to high) by discrete scale to the new resources.

Hard trust by contract was proposed by [9]. It overcomes the soft trust solutions. While soft trust evaluates trust by judgments, hard trust invokes the service before the interaction. This method suffers the demerits of maintaining the registry for providers list and to maintain the trust levels, for all the available service that dampens memory efficiency. A proactive reputation for rating new resources and to attend the cold start issue was proposed by [10]. P2P reputation model was carried out, in which, to generate the reputation ratings, proactive initiation for interaction among peers was proposed. Endorsement based web service selection was proposed by [11]. This method is used to endorse the new services by trusted participant in the system. Difficulty in assessing the newcomer is not cited. Trust bootstrapping based on ontology has been proposed in [12]. It addresses the problem of initial trust by means of peer review process. Though the method evaluates the trust during bootload of web services, it involves coordination amongst the available peers. Coordinating is a task to be handled against some cost. Predefined communities evaluate the trust. In another case, trust can be assigned with some initial assumption [12]. Both cases do not attend the problem of trust under uncertainty.

The problem of initial trust by trusted recommender (community based) was attended by [13]. Updating the recommenders trust level seize considerable amount of computation time, thus directly reflecting the computational complexity at initial level. Also, reliability of trust from external entity is an issue. A novel method by introducing the concept of new metrics for trust was proposed in [14]. Their method suffers from the problem of segregating the resource metrics, based on context. Computation time is considerable and also the method has the drawback of assigning some default initial value. The methods discussed above, addresses the problem of initial trust or trust before interaction. Several demerits can be quoted from the above list, for instance, assigning default value for unrated (new) resources; if genuine resources are assigned a low trust value, probability of provisioning of resources may get stifled. At the other end, if a nongenuine resource gets assigned a high trust value, then the outcome may lead to disastrous outage. Gathering reputation or recommendation for unexplored resources is uneasy as the resources behavioural attributes are uncertain. Problem of exploring potential cloud resources under uncertain circumstances is to be considered as an earnest issue. Most cited works do the task of evaluating the trust in certainty. Under uncertain circumstances, random assignment of trust values, as adopted by the previous works, may not be fair with respect to genuine resources. Uncertain resource trust levels and uncertain user behaviour makes beginning uneasy. Cloud computing includes new resources (without pretrust evaluation) and it is obvious that new users are inevitable. Hence evaluating trust under complete uncertainty in a fair manner improves the number of potential resources at the initial stage. Furthermore, the problem of cold start and whitewashing are supposed to be addressed. Thereby, the total number of resources available for service can be increased. In general, as said in [15] “establishing trust in cloud systems requires two mutually dependent elements.” Whether it is boot level or higher level, both entities must cooperate for trust.

## 3. System Model and Methodology

**Figure 1:**
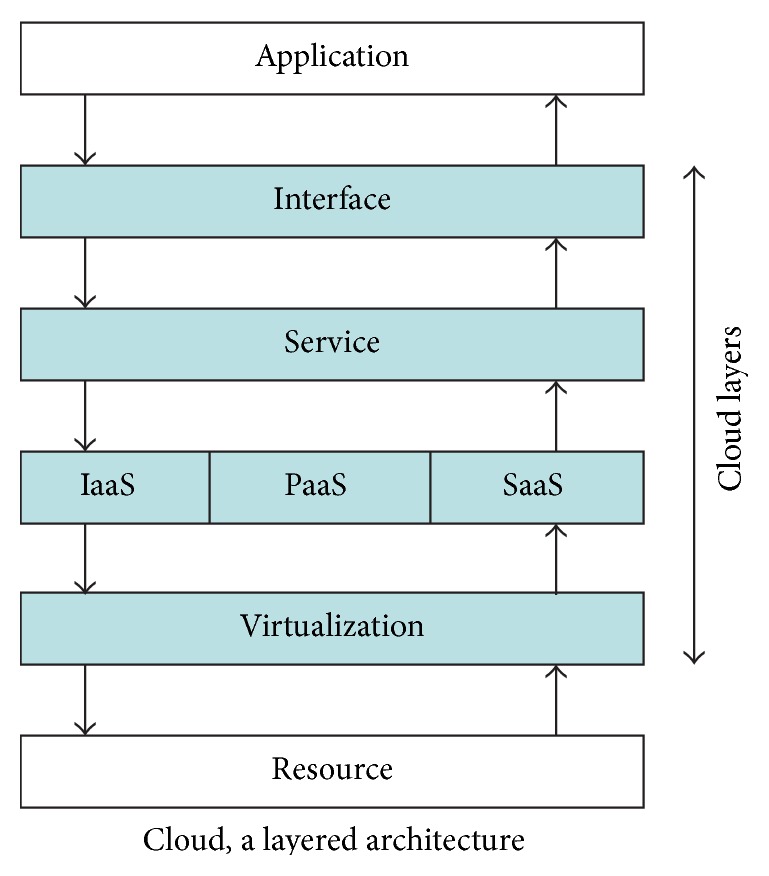
Proposed layered model.

Objective of the proposed method is to present a novel model to evaluate trust for naive entities in cloud environment with an unbiased manner. Also, the proposed method neutrally evaluates trust for both user and resource provider. Outline of the cloud model adopted in this work is depicted pictorially in Figure 1. Layered approach paves easy deployment of the versatile services within the cloud. Highlighted boxes are the core in-house layers to the cloud. User and resource are the end layers. Interface collects requirements from the user and displays potential resource providers list to the user. Service layer carries the task of scheduling, load balancing, SLA creation, monitoring, and trust managing. Service layer is elaborately discussed in later sections. Based on the selected type of service (IaaS, Paas, and SaaS), appropriate service layer proceeds the task further. Virtualization is the closely associated layer with physical resources and the actual service deployment done by this layer.

**Figure 2:**
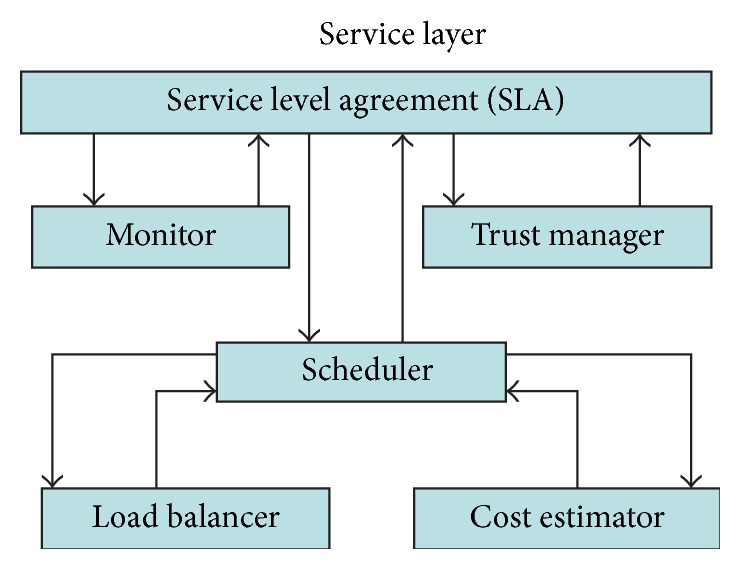
Service layer.

Detailed service layer illustrated pictorially in Figure 2. SLA (service level agreement) ensures two parties (user and provider) and furnishes actual statements that are available before the service begins and recorded for future correspondence. The trust manager updates the level of trust for an entity against another by applying* Nash equilibrium* by forming a game structure between user and provider. The monitor continuously monitors the SLA for any violation and predefined accord. To play a game and to attain Nash equilibrium, certain parameters are mandatory with respect to trust relationship. These parameters, namely, risk, belief, disbelief, and uncertainty, are highlighted in the next section.

### 3.1. Parameter Description

*Risk.* Whenever the notion of trust is discussed, risk is the associated parameter of interest. Risk is an integral part of trust. The relationship between trust and risk is highlighted [16]. It states that trust and risk are in complementary relation; that is, the higher the trust, the lower the risk and vice-versa. Risk of the provider's resources is under lime lights, when accessing the services. Assessing the risk involved helps in evaluating trust of an entity. Based on risk, user can be segregated into three broader categories:

1. risk seeking;
2. risk neutral;
3. risk aversion.

This work considers risk as the key parameter for evaluating trust. An application is segregated along with risk as risk seeking application, risk neutral application, and risk averse application. Once the application is segregated, the next measure is to estimate the risk associated with it. In fact, risk estimation becomes easier after segregating the type of application. 

*Belief.* Belief is the degree of confidence expressed as objective and subjective, with respect to entity and time. Belief is a dynamic entity, which changes with respect to time and context in any computational paradigm. Cloud is a dynamic environment, wherein the belief of an entity over others changes frequently. The proposed method uses belief as a subjective logic over cloud user's perception to cloud resource provider. The cloud resource provider also expresses it as the same for cloud users. Josang and Ismail present models and methods to assign weights for belief [2]. 

*Disbelief.* Disbelief is the degree of doubt over an entity. In belief, degree of disbelief changes with respect to time and context. Disbelief refers to the negative consequences expected. 

*Uncertainty.* When there is no previous interaction with an entity, uncertainty emerges over its trustworthiness. Usually, resources performing the very first transaction of any kind of services face this challenge. Resources with uncertain specifications are normally not considered to avoid any future loss by the users as well as the service providers (in case of higher transaction values). 

Belief, disbelief, and uncertainty together form a belief additivity function represented in [2]. Uncertainty can be addressed by means of substituting some probability value known as uncertain probability. User's opinion is represented as subjective belief. Furthermore Josang and Ismail state that* the sum of belief, disbelief, and uncertainty equals one* [2]. This is mathematically represented as

(1) $$\begin{array}{c} b\left(\;x\;\right)+d\left(\;x\;\right)+u\left(\;x\;\right)=1, \end{array}$$

where

 
*b*(*x*) is the belief over entity *x*;
 
*d*(*x*) is the disbelief over entity *x*;
 
*u*(*x*) is the uncertainty over entity *x*;

thus, any two entities are sufficient for expressing trust.

### 3.2. Methodology

This work focuses on* game theory* as a major framing structure, as it forms a game between user and service provider through a bimatrix. Bimatrix game formation helps us to achieve a service level agreement between the user and the provider through Nash equilibrium. Since 2-player Nash equilibrium falls under PPAD-complete [7], time complexity is handled more efficiently than other methods. Nash defines game theory as a* study of strategic decision making. Specifically, it is the study of mathematical models of conflict and cooperation between intelligent rational decision makers* [17]. Game theory provides a mathematical way of solving situation conflicts. Also, it helps to analyze the efficiency of decision making where interdependence between entities exists. At least in zero-sum games, it frames an experimental computing means to reach an optimal strategy. General elements of a game include player, action, strategy, result, information, equilibrium, and payoff. A single player will have his/her own strategy derived from chosen action and the payoff for the action from the given information.

*Player.* A player is rational decision maker and intends to maximize his payoff. 

*Payoff.* It is the expected outcome for a player. 

*Strategy.* A strategy (A) means the action selected by a player in each iteration. *S* = {*s*_1_, *s*_2_,…, *s*_*n*_}. 

*Strategy Profile.* It is a set of strategies selected by players involved in each iteration. 

*Equilibrium.* It is a strategy profile equally poised for all players. 

*Nash Equilibrium (NE).* NE is a solution concept of a game involving two or more players, in which each player is assumed to know the equilibrium strategies of the other players, and no player has anything to gain by changing only his or her own strategy unilaterally [18]. 

To simplify further, Nash equilibrium is a strategy agreed between two players where none of the players has the chance to deviate from the agreement to benefit unilaterally.

Let us consider (*S*, *F*) as a game with *n* players; here *s*_*j*_ is considered as a strategy set for player *j*. As discussed earlier *S* = {*s*_1_, *s*_2_,…, *s*_*n*_} is the set of strategy profiles with *S*_*u*_ as set of strategy profile for user and *S*_*p*_ as strategy profile for provider and *F* is the payoff function for *x* ∈ *S* with *F*_*u*_ as payoff function for user and *F*_*p*_ as payoff function for provider. In this scenario, *S*_*u*_, *S*_*p*_ ∈ *S* and *F*_*u*_, *F*_*p*_ ∈ *F* form the set (*S*, *F*). Let *x*_*j*_ be a strategy profile for player *j* and let *β*_−*i*_ be a strategy profile of all players except for player *i*. When each player *i* ∈ {1,2,…, *n*} chooses strategy *x*_*i*_ resulting in strategy profile *x* = {*x*_1_, *x*_2_,…, *x*_*n*_} then player *j* obtains payoff *f*_*j*(*x*)_. A strategy profile *x* ^*∗*^ ∈ *S* is Nash equilibrium (NE) if no unilateral deviation by single player is selfishly profitable by particular strategy; that is,

(2) $$\begin{array}{c} \forall i,x_{i}\in S_{i}:f_{i}\left(\;{x_{i}}^{*},{x_{-i}}^{*}\;\right). \end{array}$$

When the inequality above holds strictly (with > instead of ≥) for all players and all feasible alternative strategies, then the equilibrium is said to be a strict Nash equilibrium. If, for some players, there is exact equality between *x*_*i*_^*∗*^ and some other strategy in the set *S*, then the equilibrium is said to be a weak Nash equilibrium. Thus, a game between a user and a provider makes SLA achieve Nash equilibrium through a bimatrix game resulting in trust by adopting the above discussed parameters. Nash equilibrium is applicable for one-time game only and considers players rationality. This characteristic of NE helps to coordinate two players for optimal payoff.

## 4. Game Model

The proposed work extends (1) from a single linear equation to two-dimensional matrix representations (a bimatrix game) as a primary objective. Equation (1) is used to express the parameters like belief, disbelief, and uncertainty by a single user only. This gives the chance for single user to express the weights of desired attributes, whereas in a game model minimum of two users gets involved. Hence, a minimal matrix representation is required here. While a cloud user's perception forms one dimension, resource provider forms another dimension and the two are equally poised to infuse fairness towards estimation of trust. Since, the bimatrix game is formed with mixed profile strategies, equal chance of both cloud user and resource provider is entitled through proper trust management activity. Moreover, one-time games are applicable for naïve entities alone. Thus (1) extended as a game arena between a user and a service provider is represented in Table 1.

Table 1 enclosed in SLA of the proposed architecture obtains perception values from the cloud user and the service provider with respect to (1).

It is mandated by the SLA for both cloud users and the service providers to assess the type of application/resource level risk involved prior to assigning values for the belief, disbelief, and uncertainty. In fact, as per the design of the proposed work, only after considering the appropriate risk level, a cloud user or service provider can start assigning value to belief, disbelief, and uncertainty. Thus, the type of risk is mapped with the type of application/resource as follows. A sample of risk type and associated cloud user's application and resource provider's resources are highlighted in the following.

*Risk Aversion (Type of Risk), for Example, Real Time Application.* For Example, there are several mission critical applications, which require cloud environment, where a video streaming kind of application could tolerate the risk of unavailability for a few seconds. At the other end, some mission critical resources like bank transaction like services cannot tolerate the risk of unavailability.

*Risk Neutral (Type of Risk), for Example, Free Mobile Apps.* Several applications do exist with the requirement of even more time flexible services under cloud environment. For example, static pages of portals can tolerate the risk of delay for a certain time period. At the other end service providers hosting offline applications under cloud shall not tolerate the risk slackness in catering the services.

*Risk Seeking (Type of Risk), for Example, Time Invariant Application.* Applications like time insensitive streaming of multimedia applications and certain applications like offline downloading can be performed even with risk of loss during the transaction. Service providers hosting offline time insensitive and noncasual applications under cloud can tolerate the risk of delay in times.

**Figure 3:**
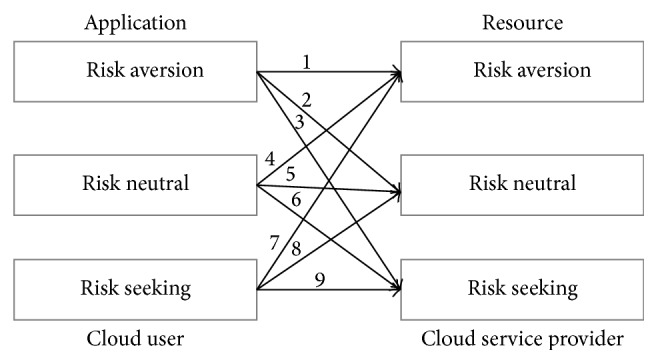
Mapping of cloud users risk to resource providers risk.

A prospective cloud user to resource provider mapping is shown in Figure 3. It shows mapping numbers from 1–9 for three types of risk from cloud users to resource providers. Here mappings 1, 5, and 9 are straight mapping. Here risk averse kind of applications are mapped to risk averse kind of resources, risk neutral applications are mapped with risk neutral resources, and risk seeking applications are mapped with risk seeking resources. Thus, level of belief can be set at a high level for these direct mapping theoretically, say, more than 66%. For practical reasons, level of percentage is restricted to kind of mapping and a cap of 85% is considered. Since more than 85% [15] of value may end up with dominated strategy in calculating the equilibrium, such percentage forces other values to marginally lower the chances of Nash equilibrium. Hence, threshold of 85% is considered. Mappings 2, 4, 6, and 8 are considered as midlevel belief, since the nature of neutral risk is involved in either of the entities. Thus, it can ends up in the range of 33% to 66%, and remaining mappings 3 and 7 are considered as low level of belief.

In this mapping, 33% of value is considered as the grade separator for one kind of belief value to another, because for a risk neutral application, 33% of belief value can be applied for all the three types of risk. Here, high level belief values are belief (*b*), midlevel beliefs are uncertain (*u*), and low level beliefs are disbelief (*d*).

Apart from these types of segregation considered by players, Jensen's inequality [19] is used by players to averse the risk. It states that

(3) $$\begin{array}{c} \int v\left(\;x\;\right)df\left(x\right)\leq v\left(\;\int df\left(\;x\;\right)\;\right), \end{array}$$

where

 
*v*(*x*) is gain utility for *x*;
 
*f*(*x*) is nondegenerate value for *x*;
 ∫*x* *dF*(*x*) is expected value.

*f*(·) is a cumulative distribution function; thus the continuous utility value is integrated with respect to nondegenerate value to estimate the outcome. On the other hand expected utility of *x* is calculated and compared by considering the type of risk, for risk aversion (3) is applied to averse the risk. Since risk seeking is inverse of the risk averse scenario, (3) can be converted to risk seeking as

(4) $$\begin{array}{c} \int v\left(\;x\;\right)df\left(x\right)\geq v\left(\;\int df\left(\;x\;\right)\;\right) \end{array}$$

and, for risk neutral, as

(5) $$\begin{array}{c} \int v\left(\;x\;\right)df\left(x\right)=v\left(\;\int df\left(\;x\;\right)\;\right). \end{array}$$

After assigning the values from either end of the cloud, SLA forms the game between users and providers. A model SLA as a game is given in Table 2.

### 4.1. Solving for Nash Equilibrium (NE)

The present work uses the algorithm of [18] for attaining Nash equilibrium, over Lemke-Howson algorithm. Thus, we use four steps to achieve it which are as follows:

1. tableau formation;
2. identifying entering and leaving variable;
3. result.

**Step 1 .**

By identifying the strictly dominated strategies from the mixed profile strategies, fairness can be preserved. Fairness helps to proceed the game equally poised for both cloud users and service provider. This is achieved by applying row dominance rule and column dominance rule, respectively.

**Step 2 .**

Tableau formation of the available mixed profile strategies after applying dominance helps to identify Nash equilibrium from Step 3.

**Step 3 .**

Pivoting elements are identified arbitrarily from cloud users or cloud provider's tableau formed in the previous step and min-ratio rule is applied for appropriately identifying the entering and leaving variables. The step is repeated until a unique variable is obtained as the winner.

**Step 4 .**

From the appropriate variable's row, Nash equilibrium is achieved from the previous step.

One advantage from the above cited method to this work is that need for normalization shall be optional. Normalization is applied only when the resulting Nash equilibrium deviates from the listed strategies. That is to identify the nearby strategy from the tableau and not for normalizing to the value of (1) as in [18]. Also, the method fixes both entities to be in a single strategy throughout the transaction.

### 4.2. Algorithm

See Algorithm 1.

## 5. Results and Discussion

The core objective of this work is to frame a model for cloud environment at bootstrapping level. This work was experimentally evaluated by simulation using cloudsim and studied the performance. Test bed consists of the attributes shown in Table 3.

**Figure 4:**
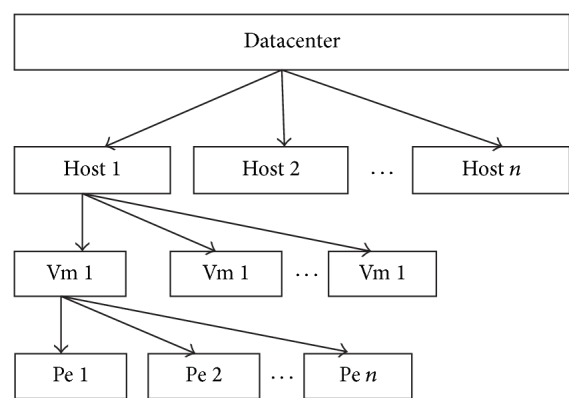
Cloudsim hierarchy.

As defined in [20] and depicted in Figure 4, datacenter is at the higher level of abstraction in the service/resource provider's end. This forms the highest external interface from provider's part. Host comes under the datacenter and aggregates the virtual machines (VM's). A datacenter may contain “*n*” numbers of host bounded by its capacity; virtual machines are the actual boxes that replicate the actual physical boxes. A host may have “*n*” number of VM's, which are created according to the requirements. Final level of hierarchy forms the processing elements (pe's). Pe's are similar to actual processors, which performs computing inside the VM's. Multiple pe's of a single machine can be made as homogeneous and/or heterogeneous clusters. The proposed work is compared with the existing approaches like hard trust and metric based trust.  Table 4 summarizes the three methods in detail.

### 5.1. Availability

**Figure 5:**
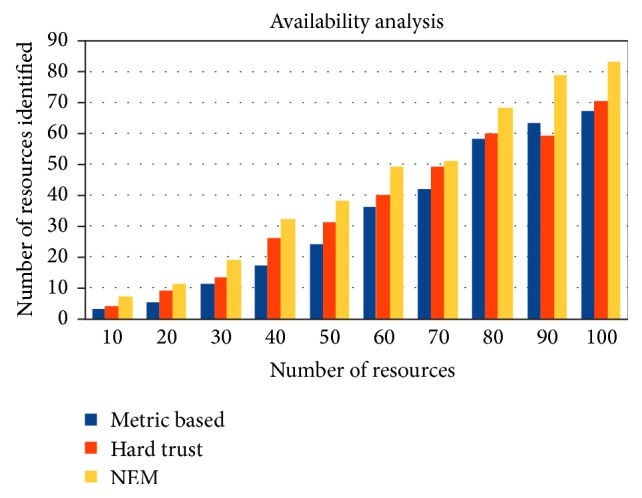
Trusted resources identified.

Identification of trusted resources at the bootloading level is the prime focus of this work. 10 to 100 numbers of resource providers were considered for simulation setup in cloudsim environment. Results reveal that Nash equilibrium method (NEM) out-performs hard trust and metric based trust bootstrapping mechanisms for identifying more number of resources at the boot level. Hard trust based bootstrapping evaluates the trust for a given resource through semantics identified by the evaluator (user/provider). Since semantics need to be stored in the form of a database, considerable amount of time is required for retrieving the database. Furthermore the semantics for fresh dimensions are not easy for newer context resources. Metrics based trust evaluation indirectly cites risk involved in entities. Attributes may not be defined for the entire resource context; sometimes it may be redundant as well. Availability of fresh and genuine resources is sometimes not evaluated as shown in Figure 5, due to these reasons. Trustworthy resources identification by NEM involves direct risk based metric as provided by the user and provider. In spite of being so close to hard trust based method in few instances, marginally NEM outperforms those instances. Overall NEM identifies more genuine resources than other cited methods. From the results it is clear that NEM performs 12% to 40% better than metric based trust and achieves an improvement of 3% to 30% over hard trust. Average betterment of NEM over metric based trust is slightly higher than 24% and 15% over hard trust. The more the number of available resources at the bootload level, the more the number of services and, hence, the better the service ratio and the higher the* throughput*.

### 5.2. Undecidability

**Figure 6:**
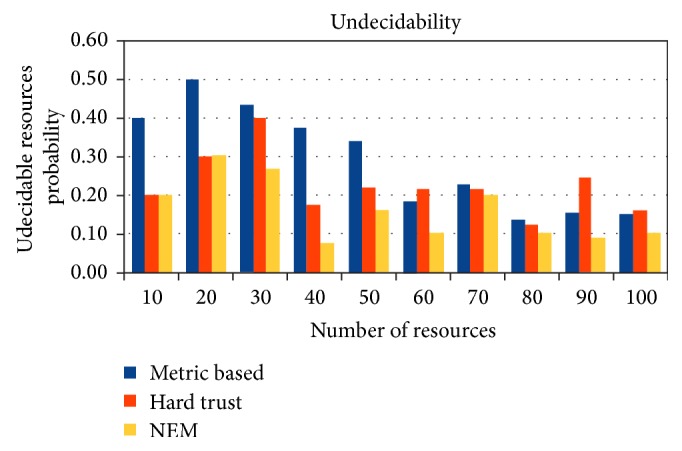
Unevaluated resources.

Methods cited in the literature over multiple instances fail to evaluate the resources to be trusted or untrusted for certain resources. As depicted from the experiments conducted against hard trust, metric based, and NEM methods, there are several instances where trust evaluation algorithms were terminated with undecidability. Metrics based evaluation will no longer hold, if the requirement does not match the resources with specified metrics. There are instances where the metrics carry null value and thus paves the way for undecidability. Hard trust method is paralyzed when no matching semantics were mapped. NEM also suffers from undecidability, when the risk parameter is completely uncertain from user and/or provider.  Figure 6 shows at least 3% to 17% lesser undecidability with respect to metric based evaluation, 16% lesser undecidability with respect to hard trust evaluations, and an average of 13% and 7% lesser undecidability, respectively. It can be observed that the lesser the number of undecidable resources, the more the number of evaluations.

### 5.3. Erroneous Evaluation

**Figure 7:**
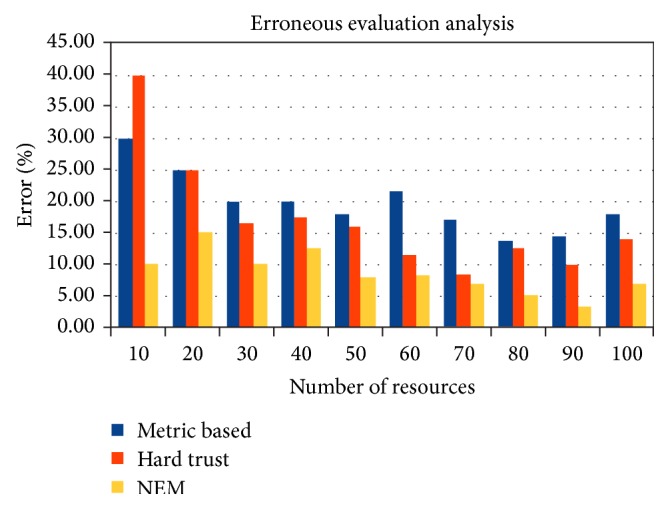
Time taken for trust evaluation.

The experiment also shows (Figure 7) the erroneous evaluation of resources, where untrustworthy resources are reported as trustworthy. Several untrusted resources were generated for simulation purpose and tested with all the three methods. In each and every set of resources, from 10 to 100, 0% to 100% of untrustworthy resources were incorporated and the results shown above are the aggregation over the instances ranging from 10 to 100 set of resources. While all the above algorithms do falsely evaluate the resources as trustworthy, attenuation of errors in number is achieved through proposed NEM method. While the highest number of erroneous evaluation is reported in hard trust method with 40%, only 15% was reported in NEM and 30% in metric based. Maximum of 97% reliability is achieved through NEM and minimum is 85%, while 86% and 70% in metric based evaluation and 91% and 60% in hard trust evaluation approach. Thus, lesser erroneous evaluation improves reliability of the evaluation methods.

### 5.4. Accuracy

**Figure 8:**
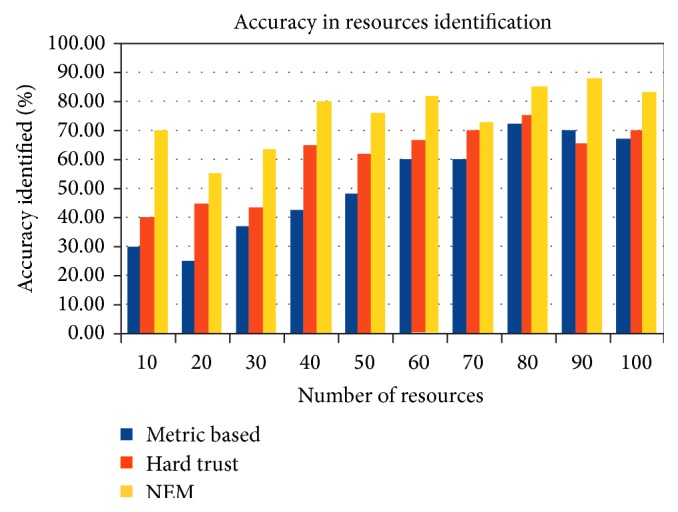
Trusted resources accuracy in percentage (%).

Figure 8 depicts that a higher level of accuracy is achieved by the NEM method with more than 80% accuracy in 50% of the instances simulated. Simulation results in varying number of resources from 10 to 100. Metric based method achieves more than 70%, while metric based method end up with just above 60%.

### 5.5. Overhead

**Figure 9:**
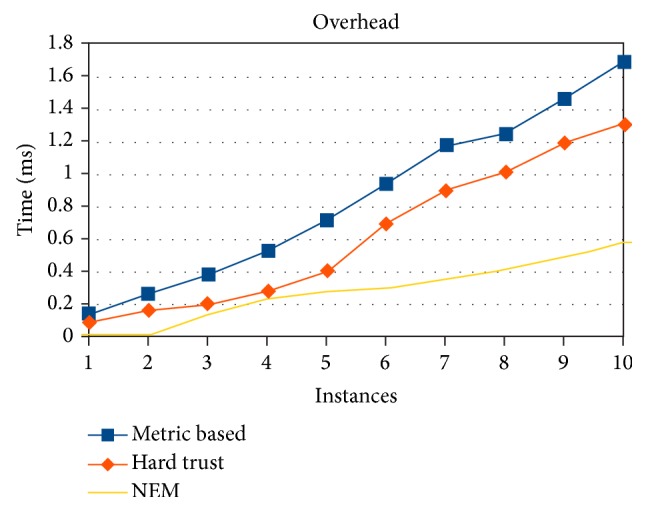
Trusted resources error in percentage (%).

To carry out the evaluation of trust over a given set of new resources, certain tolerable overheads can be incorporated. One such overhead is time taken for deciding the resource to be trustworthy or untrustworthy. Experiments conducted exemplifies (Figure 9) that overhead is lesser in NEM than metric based and hard trust based methods. Evaluation of more number of parameters and its associated complexities will result in high time consumption for metric based method. Depending on database like structure for maintaining and retrieving semantic aggravates the complexity in hard trust. Due to the mapping techniques discussed in previous section, lesser amount of time for evaluation is achieved in simulating the specified number of resources.

## 6. Conclusion

This work proposed a model for evaluating trust for first-time user/provider in cloud environment. It can be further extended to any uncertain environment. Apart from modelling the problem, simulation was also carried out to validate the model. The proposed method shows improvement in evaluation with respect to time, reliability, and erroneous evaluation. Cold start problem were addressed to a larger extent by covering maximum number of resources. Risk is studied as a parameter of direct involvement by user and service provider. This improves the efficiency by increasing throughput, availability, and reliability. At the same time risk of erroneous evaluation is attenuated to greater extent by proper mapping of appropriate resource to user. Whitewashing was largely attenuated by making appropriate mapping. Nash equilibrium ensures that neither the user nor the service/resource provider overcomes the service level agreement (SLA), as the transaction on-goes.

Future direction does exist to improve the proposed method for iterated removal of dominated strategies as transaction proceeds further. Thus, a better equilibrium can be achieved by playing the game with similar set of inputs from the second time. This work can further be extended to solve the uncertain nature of applications and resources, which actually pulls down the performance.

## Figures and Tables

**Algorithm 1:**
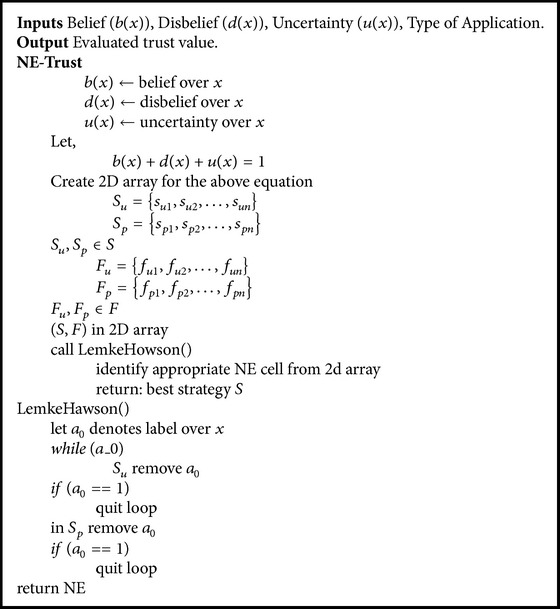
Pseudocode of the Nash equilibrium (NE) based trust model.

**Table 1:** Table form of (1).

User	Resource provider		
	Belief	Disbelief	Uncertainty
Belief	(*b*, *b*)	(*b*, *d*)	(*b*, *u*)
Disbelief	(*d*, *b*)	(*d*, *d*)	(*d*, *u*)
Uncertainty	(*u*, *b*)	(*u*, *d*)	(*u*, *u*)

**Table 2:** Table 1 with values after risk calculation.

User	Resource provider		
	Belief	Disbelief	Uncertainty
Belief	(.4, .3)	(.4, .5)	(.4, .2)
Disbelief	(.25, .3)	(.25, .5)	(.25, .2)
Uncertainty	(.35, .3)	(.35, .5)	(.35, .2)

**Table 3:** Test bed.

S. number	Entity	Quantity	Purpose
1	VM	10–100	Virtual machines
2	Cloudlet	10–10000	Job to cloud
3	User	10–1000	Service requester
4	Provider	10–10000	Service provider
5	Risk	3 types	Parameter

**Table 4:** Comparison of existing and proposed methods.

S. No	Method	Parameters	Achievements	Observations
1	Hard trust	Security features, interfaces, and service semantics.	Semantics defined for all the key attributes. Considering context level semantics.	Defining semantics for initial provider is a little complex.

2	Metric based trust	Subjective service trust metric, objective service trust metric, and trust rate.	“Services selected and trusted according to their security features, and they are discovered according to their interfaces or semantic descriptions.”	Honesty of service providers at bootstrapping level is evaluated in a lighter sense.

3	Proposed (NEM)	Risk type, application type, belief, and uncertainty.	Considering risk involved with respect to both user and service provider.	Method facilitates mutual agreement at the boot loading level.
